# Evaluating digital resources as a supplement to traditional anatomy teaching in dental education

**DOI:** 10.3389/fdmed.2026.1781318

**Published:** 2026-03-18

**Authors:** Noora Helene Thune, Anna Tostrup Kristensen, Hugo Lewi Hammer, Qalbi Khan, Tor Paaske Utheim, Amer Sehic

**Affiliations:** 1 Institute of Oral Biology, Faculty of Dentistry, University of Oslo, Oslo, Norway; 2 Department of Medical Biochemistry, Oslo University Hospital, Oslo, Norway; 3 Department of Plastic and Reconstructive Surgery, Oslo University Hospital, Oslo, Norway; 4 Department of Computer Science, Faculty of Technology, Art and Design, Oslo Metropolitan University, Oslo, Norway; 5 Inland Norway University of Applied Sciences, Elverum, Norway

**Keywords:** blended learning, dental education, digital learning, knowledge retention, oral macroscopic anatomy teaching

## Abstract

**Objective:**

Oral macroscopic anatomy forms a core component of dental education, requiring both detailed factual knowledge and the ability to recognize complex three-dimensional structures. This study evaluated whether structured digital learning supplements, introduced alongside traditional teaching, improved short-term learning outcomes and long-term knowledge retention among dental students.

**Materials and methods:**

Four student cohorts from the Faculty of Dentistry, University of Oslo, were included. Two earlier cohorts (2016 and 2017; *n* = 96) received traditional anatomy teaching, while two later cohorts (2018 and 2019; *n* = 98) also had access to structured digital supplements, including lecture recordings, stepwise digital model presentations, an animated osteology key, and high-resolution images of dissected specimens. Performance was assessed during the second-year practical examination and re-evaluated 2.5 years later during the fifth-year dissection course. Logistic regression mixed-effects models were used to compare outcomes between teaching approaches, and surveys captured student perceptions of the digital resources.

**Results:**

Students with access to digital supplements achieved markedly higher scores in the second-year practical examination across osteology and macroscopic anatomy tasks. This performance advantage persisted 2.5 years later during the fifth-year dissection course, indicating enhanced long-term retention. Survey data showed extensive use of digital materials during the second year, with students reporting that the resources improved their understanding and supported examination preparation. Although overall use declined by the fifth year, many students revisited the digital resources when they needed repetition in preparation for clinical activities, such as surgical procedures.

**Conclusion:**

Structured digital supplements meaningfully enhanced traditional teaching in oral macroscopic anatomy, improving initial learning outcomes and supporting retention well into clinical training. Digital tools provided flexible, self-paced opportunities for repetition and active recall, while hands-on work with physical models and specimens remained essential. These findings support a blended learning approach in which digital resources complement, but do not replace, practical anatomy teaching.

## Introduction

Anatomy has long been regarded as a critical keystone of medical and dental education, as a thorough understanding of the human body, including oral and maxillofacial structures, is essential for safe clinical practice (1). In dentistry, detailed knowledge of oral macroscopic anatomy provides the basis for diagnostics, surgery, restorative treatment, and complication management. Deficiencies in anatomical understanding can result in technical mistakes and patient harm, underscoring the importance of effective anatomy teaching in undergraduate training (2, 3). Traditionally, anatomy has been taught through lectures, cadaveric dissections and prosections, and practical courses using different anatomical models. In dentistry, textbooks and atlases have often been used to supplement these approaches, supporting spatial understanding and repetition. Despite its central role, however, the optimal way of teaching anatomy remains debated (4). The challenge is not only to convey factual knowledge but also to ensure its long-term retention and clinical applicability (5, 6).

In recent decades, digital resources have increasingly been integrated into anatomy education. These include lecture recordings, online atlases, interactive 3D models, mobile applications, and high-resolution images of dissected specimens. Such tools extend learning beyond the classroom, allow repeated visualization of complex structures, and accommodate diverse learning preferences (7). The introduction of these resources has coincided with the emergence of “digital native” student cohorts, who are accustomed to technology-rich learning environments (8). The role of digital resources in anatomy education, however, remains contested. Advocates argue that they offer accessible, flexible, and cost-effective learning opportunities, while critics emphasize that cadaver-based dissection provides irreplaceable three-dimensional, tactile experiences and fosters professional attitudes toward the human body (9, 10). Consequently, the balance between traditional and digital approaches is still unresolved. Moreover, while students often express a preference for digital tools, preferences may not align with the methods that best promote deep learning and knowledge retention (10).

Integrating online resources with limited course-based teaching represents a form of blended or hybrid learning (11). Studies have reported that such approaches may lead to improved learning outcomes (12, 13), although in some cases no significant differences have been observed compared with traditional face-to-face instruction (14). Online components offer several potential benefits, including cost efficiency in resource provision, reduced demands on academic staff following initial development, and enhanced flexibility by enabling students to access learning materials off campus at any time. Nevertheless, an important drawback is the possibility that greater reliance on online tools could reduce the frequency of course sessions. This may limit opportunities for peer-to-peer interaction, collaborative skills development, and direct engagement with instructors, all of which are valuable elements of anatomy education (15, 16).

The present study aimed to evaluate whether structured digital learning resources, used as supplements to traditional teaching, enhance learning outcomes and long-term knowledge retention in oral macroscopic anatomy among dental students. Specifically, we compared examination performance between cohorts receiving traditional anatomy teaching alone and cohorts with additional access to digital resources, assessing both short-term outcomes during the second-year practical examination and long-term retention 2.5 years later during the fifth-year dissection course. In addition, we explored students’ perceptions of the digital resources and their perceived value for learning, repetition, and examination preparation.

## Materials and methods

### Teaching methods in oral macroscopic anatomy

At the Faculty of Dentistry, University of Oslo, teaching of the craniofacial complex, including oral macroscopic anatomy, takes place during the second year of the five-year dental program. This module forms part of a three-month course covering anatomy and physiology of the craniofacial complex, with oral macroscopic anatomy constituting approximately six weeks. Teaching culminates in a written examination and a practical station examination, the latter providing the results analyzed in this study. Traditionally, instruction has consisted of lectures supported by PowerPoint presentations, anatomical models, and dissected wet specimens. In recent years, structured digital learning resources have been introduced as supplements to traditional teaching, including lecture recordings, stepwise digital presentations of anatomical models, and digital representations of dissected specimens. The present study was designed to compare student performance and learning experiences before and after the introduction of these digital supplements.

The course comprises 26 lectures (45 min each) and 33 h of practical courses. Attendance is recorded for both, though lectures are optional, while course participation is mandatory under a 15% absence rule. Students exceeding this threshold are not permitted to sit for the examination and must repeat the course the following year. Lectures and courses follow a structured progression, beginning with two intensive weeks of osteology, forming the foundation for subsequent topics. Teaching then proceeds through myology, angiology, and neurology, before culminating in regional or topographic anatomy, with clinical perspectives embedded throughout. The lectures address anatomy in detail, including the origins, insertions, courses of muscles, innervations, as well as the pathways and target areas of blood vessels and nerves.

**Figure 1:**
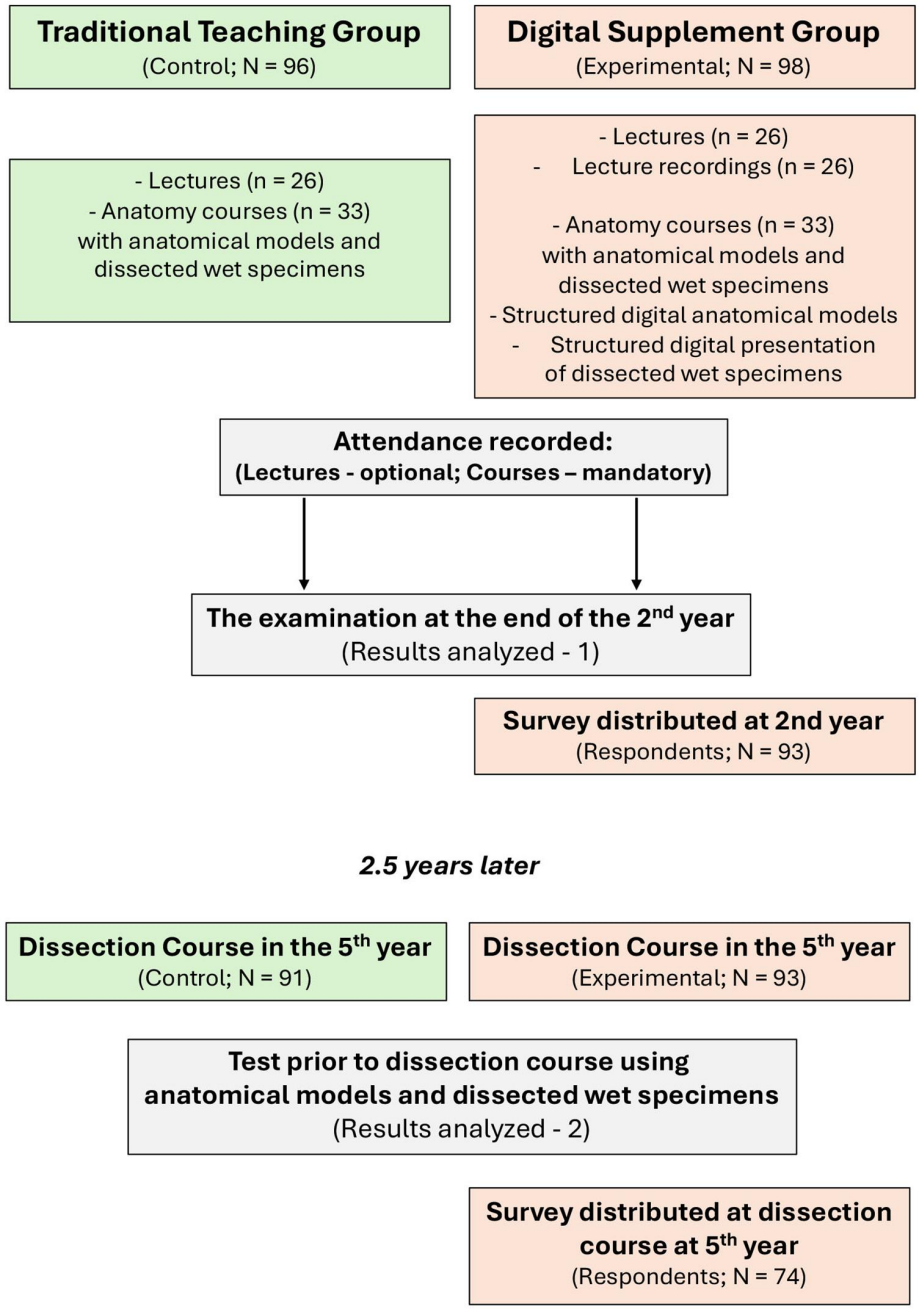
Flowchart of the study design. The *Traditional Teaching Group* (control) received lectures (*n* = 26) and anatomy courses (*n* = 33) with anatomical models and dissected wet specimens. The *Digital Supplement Group* (experimental) received the same teaching plus lecture recordings, structured digital models, and digital images of wet specimens. Both groups were evaluated at the 2nd-year examination and again 2.5 years later at the 5th-year dissection course. Surveys were distributed to the experimental group in both years. Lectures are 45 min; courses are indicated in full hours.

Practical courses are conducted in small groups, where students primarily work independently under faculty supervision. Students examine real human skulls and individual bones, 36 anatomical models, and approximately 10–15 dissected wet specimens. For the osteology component, students receive a comprehensive compendium with systematic descriptions of each individual bone, accompanied by illustrations in which key structures are indicated with pins or arrows. Students are required to correctly label these structures based on the descriptions. In addition, all anatomical structures must be identified on real bones. Each model or wet specimen is accompanied by a structured list of anatomical features that students are expected to locate and identify. The learning objective is repeated practice, enabling students to consolidate recognition and retention of anatomical structures. At this stage, students do not perform dissections themselves. However, in the fifth year, they participate in a two-day dissection course, designed to provide vertical integration and repetition of anatomy (Figure 1). At this point, students have acquired substantial clinical experience, for example in oral surgery, and are thus better positioned to contextualize and deepen their anatomical understanding.

### Student cohorts and study design

The study included four student cohorts enrolled in the dental program at the Faculty of Dentistry, University of Oslo (Figure 1). Cohorts were defined based on the teaching approach used during their second-year oral macroscopic anatomy course. Two cohorts (2016 and 2017; *n* = 96) received traditional teaching consisting of lectures and practical courses only and constituted the traditional teaching group. Two subsequent cohorts (2018 and 2019; *n* = 98) received the same traditional teaching supplemented with structured digital resources and constituted the digital supplement group. To increase representativeness and reduce the influence of cohort-specific variation, two consecutive year cohorts were included in each group rather than relying on a single cohort per teaching approach. Informed consent was obtained from all participating students prior to participation. Inclusion criteria comprised all students who completed their second-year anatomy teaching at the University of Oslo. At the fifth-year follow-up, some students were excluded because they had completed their second-year anatomy education at other European institutions before transferring to the University of Oslo, making their anatomy training non-comparable to the study cohorts. Data were collected from the second-year practical station examination and from a written anatomical identification test administered prior to the fifth-year dissection course. All examinations were corrected and validated by three of the authors to ensure consistency and reliability of scoring.

**Figure 2:**
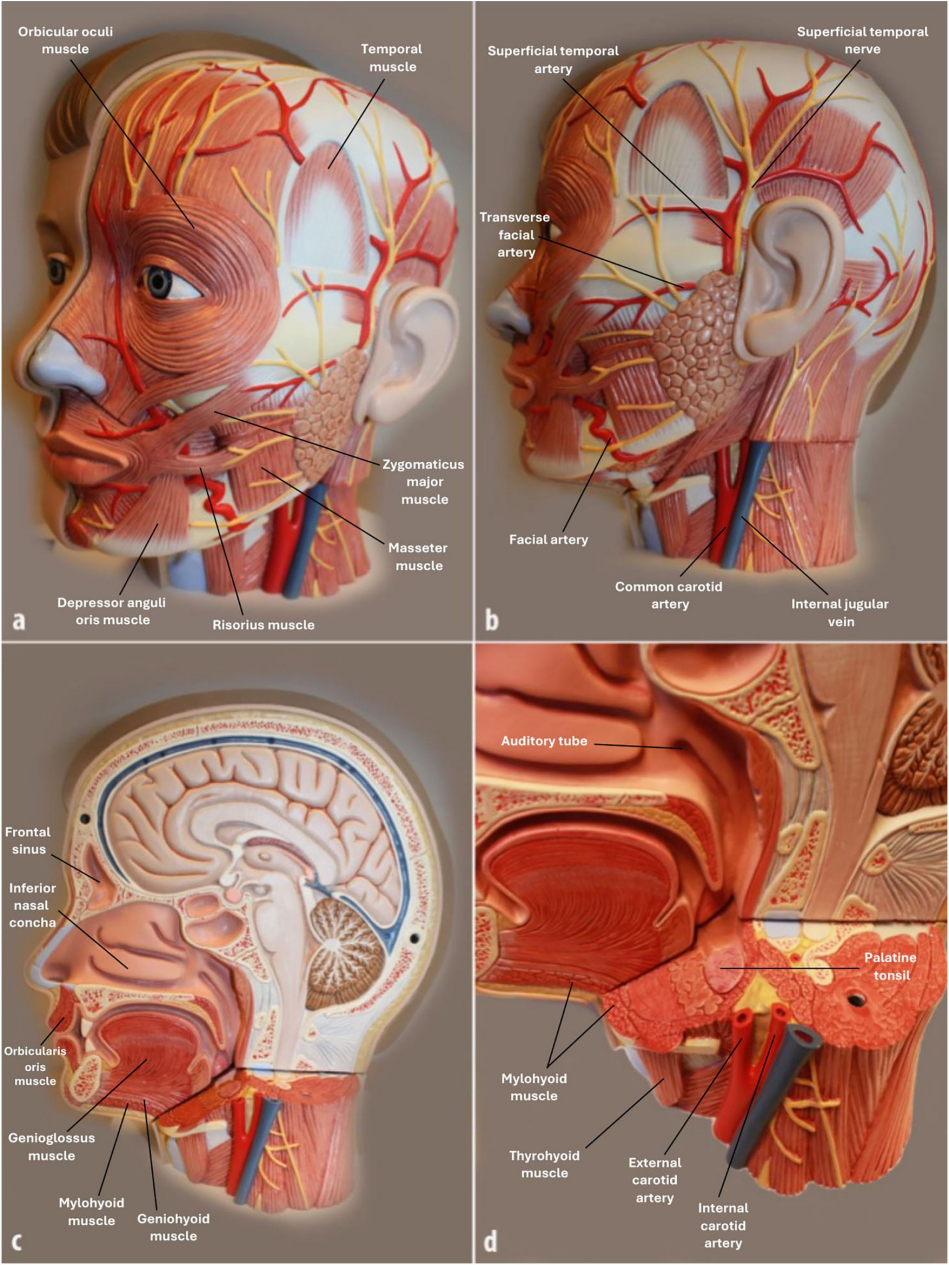
Example of anatomical models with selected marked structures used in the oral macroscopic anatomy course. Illustrated here is one of the 36 models available to students. For the digital supplement group, each model was photographed from multiple angles **(a–b)**, and all anatomical structures were systematically labeled in a stepwise PowerPoint format. In this format, a pointer first indicates the structure, and the name is revealed on the subsequent click, allowing students to actively practice identification. These resources were available both during the course and for later independent study.

**Figure 3:**
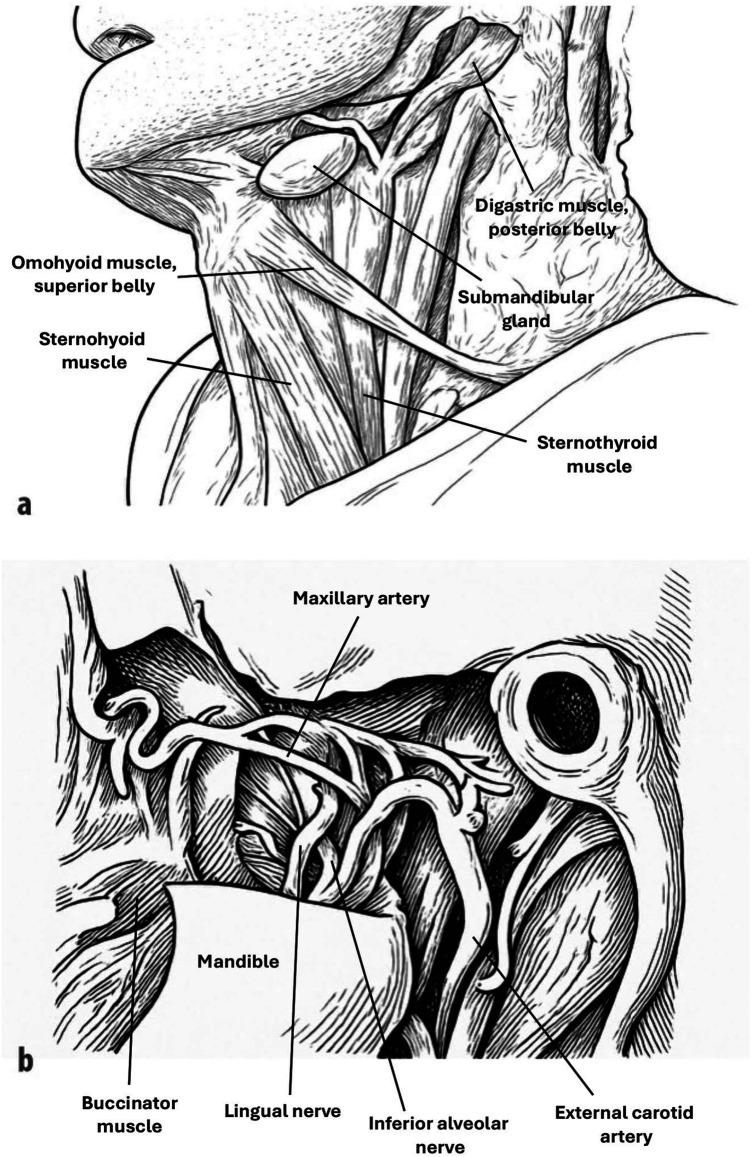
Example of schematic drawings of dissected wet specimens used in the oral macroscopic anatomy course. Shown here are two of the 25 specimens presented exclusively to the digital supplement group: **(a)** structures of the neck region below the mandible, and **(b)** the infratemporal fossa. Each specimen was photographed and illustrated from multiple perspectives, with anatomical structures systematically labeled in a stepwise PowerPoint format. In this format, the structure was first indicated with a pointer, and its name appeared with the next click, enabling students to actively practice identification. These resources were provided in addition to the regular course, both for use during teaching sessions and for later independent study.

The digital supplements included lecture recordings, an animated PowerPoint-based answer key for the osteology compendium, structured digital presentations of the 36 anatomical models (Figure 2), and high-resolution images of 25 dissected wet specimens (Figure 3). Due to institutional regulations prohibiting the publication of photographs of real wet specimens, anatomically accurate illustrations were used to closely mimic the appearance of dissected specimens (Figure 3). Each resource followed a stepwise PowerPoint format, where an anatomical structure was first indicated by a pointer, and its name was revealed upon the next click, allowing students to practice active identification both during courses and independently. All digital learning resources were made equally available to all students in the digital supplement group through the university's learning management system, ensuring fair and unrestricted access throughout the course period.

Student performance was assessed at two stages. First, results from the second-year practical station examinations were analyzed, focusing on practical identification of anatomical structures on real bones, models and dissected wet specimens (Table 1). Second, the same cohorts were re-evaluated 2.5 years later during the fifth-year dissection course, where students completed a practical test on anatomical models and dissected wet specimens prior to the course (Table 1). This design allowed for analysis of both short-term performance and long-term knowledge retention.

**Table 1:** Percentage of correct answers among 2nd- and 5th-year dental students in oral macroscopic anatomy.

Examination tasks at 2nd year	Traditional teaching group (Control; *N* = 96)	Digital supplement group (Experimental; *N* = 98)	*P-value* *
Osteology (20)	81.0%	90.8%	<0.05
Mandible (4)	87.8%	92.9%	<0.05
Maxilla (4)	78.9%	91.6%	<0.05
Sphenoid bone (4)	83.9%	91.3%	0.075
Temporal bone (4)	84.4%	93.9%	<0.05
Cranium (4)	70.3%	84.4%	<0.001
Macroscopic Anatomy (36)	70.3%	81.3%	<0.05
Photos of wet specimens (9)	72.0%	81.5%	<0.001
Wet specimens (6)	72.9%	82.1%	<0.05
Models (21)	68.8%	80.9%	0.058
Examination tasks at 5th year	*N* = 91	*N* = 93	
Macroscopic Anatomy (60)	64.2%	73.0%	<0.05
Wet specimens (15)	61.7%	73.0%	<0.001
Models (45)	65.1%	73.0%	<0.05

Examination results are presented for students taught with traditional methods (control group) and those with additional digital resources (experimental group).

* Bayesian *P*-values.

Additionally, a brief self-administered survey was distributed to students in the digital supplement group at both the second- and fifth-year stages (Table 2). The survey consisted of a small number of structured questions, all of which are presented in full in Table 2 and primarily included closed-ended (yes/no) items assessing students’ use of and perceptions regarding lecture recordings, structured digital models, and digital representations of wet specimens. The questionnaire was developed specifically for this study to capture course-related experiences and was completed voluntary by students, after which responses were collected anonymously for analysis. No additional demographic data were collected.

**Table 2:** Student evaluations of digital resources compared with traditional teaching methods in oral macroscopic anatomy.

Survey items for 2nd-year students (*N* = 93)	Yes (%)	No (%)
1. I have actively used lecture recordings as preparation and support for learning oral macroscopic anatomy.	89 (95.7)	4 (4.3)
2. Lecture recordings have been helpful in improving my understanding and learning outcomes in oral macroscopic anatomy.	76 (81.7)	17 (18.3)
3. I have used structured digital models and images of dissected wet specimens as preparation and support for learning oral macroscopic anatomy.	92 (98.9)	1 (1.1)
4. Structured digital models and images of dissected wet specimens have been helpful in improving my understanding and learning outcomes.	87 (93.5)	6 (6.5)
5. Overall, the use of digital resources (lecture recordings, structured digital models, and images of wet specimens) has contributed positively to my performance in the anatomy examination.	85 (91.4)	8 (8.6)
Survey items for 5th-year students (*N* = 74)	Yes (%)	No (%)
1. I have used lecture recordings later in my studies to review oral macroscopic anatomy.	2 (2.7)	72 (97.3)
2. I have used structured digital models and images of dissected wet specimens later in my studies to review oral macroscopic anatomy.	18 (24.3)	56 (75.7)

The survey, conducted among 2nd- and 5th-year dental students, assessed the perceived value of lecture recordings, structured digital models, and images of dissected wet specimens for supporting learning, repetition, and examination performance.

### Statistical analysis

Examination performance data from the second-year practical station examination and the fifth-year anatomical identification test were analyzed using logistic regression mixed-effects models. For each student, performance on individual examination tasks was treated as a binary outcome (correct/incorrect). Teaching method (traditional teaching or digital supplements) was included as a categorical fixed effect. To account for dependencies in the data, student ID and examination category were included as random effects. Bayesian mixed-effects models were applied to ensure stable model estimation, with weakly informative prior distributions specified for both fixed effects and random-effect variances. Model fitting was performed using the R-INLA package in R (17).

To evaluate the effect of digital supplements on examination performance, Bayesian *p*-values were calculated, defined as the posterior probability that students receiving traditional teaching performed better than those with access to digital supplements, representing evidence against the study hypothesis.

Survey data were analyzed descriptively. Given the large and consistent differences in response patterns, formal statistical testing was not considered necessary, as the results were clear and unambiguous. Survey findings are therefore presented as proportions and percentages.

## Results

A total of 194 dental students from four cohorts were included in the analyses. Two cohorts (2016 and 2017; *n* = 96) comprised the traditional teaching group, while two cohorts (2018 and 2019; *n* = 98) comprised the digital supplement group. Attendance was consistently high in both groups, with recorded participation rates of 72% in the traditional group and 76% in the digital supplement group. While lecture attendance was optional, participation in practical courses was mandatory, which likely contributed to the overall high attendance. A small number of students were lost to follow-up between the second- and fifth-year assessments, primarily due to failure in other examinations or withdrawal from the dental program. In addition, some students were excluded from the fifth-year analysis because they had completed their second-year anatomy education at institutions outside the University of Oslo and were therefore not comparable to the study cohorts. The results presented are thus based on data from the second-year practical station examination and the fifth-year dissection course, allowing assessment of both short-term performance and long-term knowledge retention.

### Second-year practical examination

At the end of the second year, students were evaluated through a practical station exam consisting of osteology and macroscopic anatomy tasks. These tasks required identification of anatomical structures on dry bones, anatomical models, dissected wet specimens, and photos of wet specimens reflecting the teaching emphasis on independent structure recognition supported by lists of required elements for each specimen and model. Overall performance showed clear differences between groups (Table 1). In osteology (20 tasks in total), students in the digital supplement group performed significantly better (*p* < 0.05), with the total score increasing from 81.0% in the traditional teaching group to 90.8% in the digital supplement group. Correct response rates rose from 87.8% to 92.9% for the mandible (*p* < 0.05), from 78.9% to 91.6% for the maxilla (*p* < 0.05), and from 70.3% to 84.4% for the cranium (*p* < 0.001).

Furthermore, macroscopic anatomy (36 tasks in total) also demonstrated significantly higher overall performance in the digital supplement group, with correct responses increasing from 71.2% to 81.5% (*p* < 0.05). The improvement was significant for both photographs of wet specimens, increasing from 72.0% to 81.5% (*p* < 0.001), and for wet specimens themselves, increasing from 72.9% to 82.1% (*p* < 0.05). Although marked improvements were also observed for anatomical models (from 68.8% to 80.9%), this difference did not reach statistical significance (*p* = 0.058).

### Fifth-year dissection course

To evaluate long-term retention, the same cohorts were tested 2.5 years later during the fifth-year dissection course. At this stage of training, students had clinical experience in surgery and other patient-based contexts, which was expected to support deeper understanding of anatomy. The test again involved structure identification on anatomical models and dissected wet specimens. Here too, the digital supplement group achieved higher results (Table 1). Across 60 macroscopic anatomy tasks, correct responses were 63.4% in the traditional teaching group compared with 73.0% in the digital supplement group (*p* < 0.05). For wet specimens, scores increased from 61.7% to 73.0% (*p* < 0.001), while for models, they improved from 65.1% to 73.0% (*p* < 0.05).

### Student experiences and perceived learning support

Student experiences with the digital learning resources were evaluated through surveys administered exclusively to the digital supplement group (Table 2). Among second-year students (*N* = 93), a large majority reported actively using lecture recordings (95.7%) and structured digital models with images of dissected wet specimens (98.9%) to support learning and preparation for the anatomy course and practical examination (*p* < 0.001). Most students indicated that these resources improved their understanding of oral macroscopic anatomy, with 81.7% endorsing lecture recordings and 93.5% endorsing digital models and specimen images as helpful learning tools. Furthermore, 91.4% of students perceived that digital resources contributed positively to their examination performance.

At the fifth-year dissection course (*N* = 74), fewer students reported continued use of digital resources later in their studies. Only 2.7% reported rewatching lecture recordings, and 24.3% indicated using digital models and images for revision. However, qualitative feedback indicated that for those who did use them, the resources were valued as a means of refreshing anatomical knowledge before the dissection course. Students emphasized that the resources helped bridge the gap between preclinical learning and clinical application, reinforcing anatomical knowledge relevant for surgical and diagnostic contexts.

## Discussion

This study examined whether structured digital learning supplements, as purpose-designed model presentations, high-resolution specimen images, an animated osteology key, and lecture recordings, enhanced students’ learning and long-term retention in oral macroscopic anatomy. When introduced alongside established teaching methods, these digital tools were associated with substantially higher performance on second-year practical examinations and continued to confer an advantage 2.5 years later during the fifth-year dissection course. Taken together, the findings suggest that digital resources, when integrated into a blended learning model, strengthen both early acquisition and long-term consolidation of complex anatomical knowledge.

Students who had access to the digital supplements performed noticeably better on osteology and macroscopic anatomy tasks in the second year. This implies that repeated, self-paced visual exposure may help learners organize intricate anatomical relationships that can be difficult to master through time-limited course sessions alone. Importantly, the performance advantage persisted during the fifth-year dissection course. Although both groups showed the expected decline in performance over time, students with early access to digital resources consistently performed better on both models and wet specimens. This suggests deeper initial encoding and more durable memory consolidation, consistent with established cognitive principles such as spaced repetition, active recall, and distributed practice (18).

Survey data reinforce this interpretation. In the second year, students reported high engagement with all digital materials and considered them valuable for understanding and exam preparation. By the fifth year, use became more selective: while few rewatched lectures, some students continued to consult the model and specimen presentations for targeted repetition before clinical activities, including surgery. This shift indicates that students perceived the digital resources as efficient tools for refreshing specific structural knowledge in preparation for clinical or surgical contexts. The continued performance advantage among the digital-supplement cohort likely reflects this combination of deeper early learning and strategic later revision (10).

The findings align with a growing body of literature demonstrating that digital tools, when well-structured and aligned with curricular goals, can enhance learning outcomes in anatomy (19). Prior studies have shown that interactive or stepwise digital resources facilitate recognition of spatial relationships and offer opportunities for repeated practice outside scheduled sessions. Markholm et al., for example, observed that video-based materials in tooth morphology improved learning progression compared with traditional resources alone (20). At the same time, the present results underscore a consistent conclusion in anatomy education research: digital tools are most effective when used as supplements rather than replacements. Hands-on learning in anatomy with physical specimens and models provides tactile, three-dimensional, and affective experiences that digital media cannot fully replicate (21). The enhanced performance and positive experiences observed here therefore support blended approaches that combine digital flexibility with the irreplaceable pedagogical value of practical anatomy teaching (22). In this context, digital resources appear particularly effective in standardizing quality, reducing variability in student exposure, and allowing individualized repetition without diminishing the importance of in-person teaching.

Several educational mechanisms may account for the observed effects. The stepwise structure of the digital presentations required students to identify anatomical features before receiving feedback, promoting retrieval practice, which is known to strengthen long-term memory retention (23). Students could revisit complex structures repeatedly and at their own pace, supporting distributed learning and deeper consolidation. High-quality visuals and clear labeling likely reduced extraneous cognitive load, enabling learners to focus on essential anatomical relationships. Moreover, students who engaged with the material beforehand may have arrived at practical sessions better prepared, making more efficient use of faculty guidance and laboratory time. These mechanisms, taken together, align with theoretical frameworks in cognitive psychology and empirical findings in related studies examining digital resources in anatomy (23).

Several implications for anatomy education emerge from this work. Digital tools can meaningfully enhance early learning, especially for complex structures where teaching time is limited. Their benefits extend into later stages of training, supporting long-term retention that is essential for safe clinical practice (24). Students value the flexibility, clarity, and self-paced nature of digital materials, particularly during intense learning periods (25, 26). Moreover, digital supplements may help standardize teaching quality across cohorts and reduce inequities associated with variability in specimen availability or attendance. Blended learning approaches therefore appear optimal, leveraging the strengths of both digital and tactile modalities. For dental education specifically, where macroscopic anatomy directly informs clinical procedures in oral surgery, prosthodontics, and radiological interpretation, strong early anatomical knowledge is critical (1). This study suggests that structured digital materials can help students establish this foundation more effectively.

### Limitations

The present study has some limitations that should be acknowledged. First, although the title refers to *digital resources*, the digital component evaluated in this study consisted primarily of structured 3D models rather than a broad range of digital learning tools. Therefore, the findings should be interpreted in the context of 3D digital anatomy resources specifically and not generalized to all forms of digital educational materials.

The non-randomized cohort design introduces the possibility of confounding, even though teaching content, examinations, and learning objectives were highly stable across years at our faculty. Importantly, the data are not paired, meaning that the second-year and fifth-year results do not originate from the same individual students. While this limits assessment of true within-person knowledge retention, cohort characteristics at the University of Oslo are typically consistent from year to year, and teaching methods are highly standardized, strengthening comparability between groups.

The single-institution setting may reduce generalizability to other educational environments. In addition, the extent of individual student engagement with each digital component was not quantified, making it difficult to determine which features contributed most to learning outcomes. Survey data were collected only from the digital-supplement group, preventing direct comparison with students taught exclusively through traditional methods. Finally, because the digital materials were used strictly as supplements, the findings cannot be extrapolated to fully digital anatomy curricula.

### Future perspectives

Future research should explore which elements of the digital resources have the greatest impact on learning, using usage data or similar methods to understand how students engage with them and how their design can best support long-term retention. Comparative studies across multiple institutions would help determine whether the observed benefits generalize across varied curricular models. Further research should also explore how digital resources can be aligned with principles of durable memory formation, such as repeated retrieval, spaced learning, and multimodal reinforcement. Integrating digital anatomy materials with clinical cases, radiographic images, or surgical videos may further strengthen the link between preclinical learning and clinical application (27).

## Conclusion

This study demonstrates that structured digital supplements significantly enhance traditional teaching in oral macroscopic anatomy, improving early performance and supporting long-term retention well into clinical training. Students valued the clarity and flexibility of the digital materials, particularly during the demanding second-year anatomy module. Although digital tools cannot replace the spatial and tactile learning provided by physical models and wet specimens, they serve as powerful complements in a blended curriculum. The present findings support continued development and integration of structured digital resources to optimize learning, strengthen durable anatomical understanding, and better prepare dental students for the clinical demands of their profession.

## Data Availability

The raw data supporting the conclusions of this article will be made available by the authors, without undue reservation.
